# What happens in the brain of meditators when perception changes but not the stimulus?

**DOI:** 10.1371/journal.pone.0223843

**Published:** 2019-10-24

**Authors:** Jürgen Kornmeier, Evelyn. Friedel, Lukas Hecker, Stefan Schmidt, Marc Wittmann

**Affiliations:** 1 Institute for Frontier Areas of Psychology and Mental Health, Freiburg, Germany; 2 Department of Psychiatry and Psychotherapy, Medical Center, University of Freiburg, Freiburg, Germany; 3 Faculty of Medicine, University of Freiburg, Freiburg, Germany; 4 Eye Center, Medical Center, University of Freiburg, Freiburg, Germany; 5 Department of Psychosomatic Medicine and Psychotherapy, Medical Center, University of Freiburg, Freiburg, Germany; Psychologische Hochschule Berlin, GERMANY

## Abstract

During the observation of an ambiguous figure our perception alternates between mutually exclusive interpretations, although the stimulus itself remains unchanged. The rate of these endogenous reversals has been discussed as reflecting basic aspects of endogenous brain dynamics. Recent evidence indicates that extensive meditation practice evokes long-term functional and anatomic changes in the brain, also affecting the endogenous brain dynamics. As one of several consequences the rate of perceptual reversals during ambiguous figure perception decreases. In the present study we compared EEG-correlates of endogenous reversals of ambiguous figures between meditators and non-meditating controls in order to better understand timing and brain locations of this altered endogenous brain dynamics. A well-established EEG paradigm was used to measure the neural processes underlying endogenous perceptual reversals of ambiguous figures with high temporal precision. We compared reversal-related ERPs between experienced meditators and non-meditating controls. For both groups we found highly similar chains of reversal-related ERPs, starting early in visual areas, therewith replicating previous findings from the literature. Meditators, however, showed an additional frontal ERP signature already 160 ms after stimulus onset (Frontal Negativity). We interpret the additional, meditation-specific ERP results as evidence that extensive meditation practice provides control of frontal brain areas over early sensory processing steps. This may allow meditators to overcome phylogenetically evolved perceptual and attentional processing automatisms.

## Introduction

**Fig 1:**
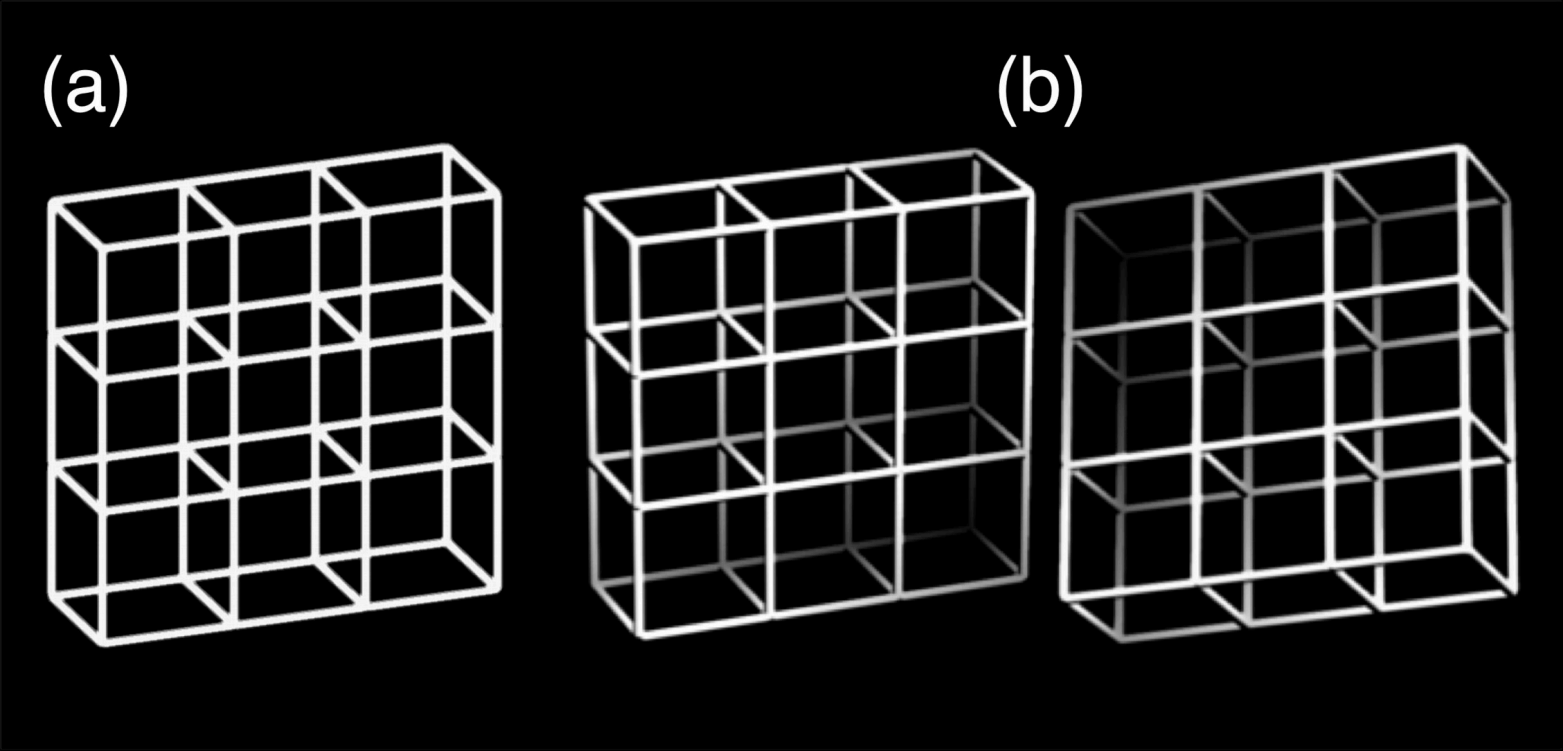
Stimuli. (a) Ambiguous Necker lattice, a combination of nine Necker cubes. (b) Disambiguated lattice variants, representing the two most probable interpretations of the Necker lattice.

During observation of an ambiguous figure, like the famous Necker cube [1], our perception becomes unstable and reverses spontaneously between mutually exclusive interpretations (Fig 1). We can volitionally control this perceptual dynamics to some degree but we cannot prevent reversals entirely [2–4]. The phenomenon of multistable perception occurs with stimuli from very different visual “categories”, like 3D perception (Necker cube), perception of motion (e.g. the stroboscopic alternative motion stimulus, [5]), during the segregation of figure and ground (e.g. the Vase-Face figure, [6]) or during binocular rivalry, i.e. when the two eyes see different images (e.g. [7,8]). Multistable perception exists also in other modalities like audition [9,10] or touch [11–13].

The topic of multistable perception has fascinated researchers from different disciplines for decades and is discussed in a variety of contexts, like decision making (e.g. [14–16]), creativity [17,18], quantum cognition [19–22], time perception and temporal integration mechanisms [23–25], as well as normal [16,26,27] and altered states of consciousness [28,29], and psychiatry [30–32].

Ambiguous figures are paradigmatic for research on basic principles of perception, awareness, and the underlying neural representations for the following reason: The information available to our senses is a priori restricted, noisy and to varying degrees ambiguous. At every wakeful moment our perceptual system needs to disambiguate the available sensory information in order to construct the most probable perceptual interpretation, as already emphasized by Helmholtz, Wheatstone and others [33–35]. Ambiguous stimuli are extreme examples for this inherent perceptual problem because most often two interpretations are about equally probable (both close to 50%) and perceptual decisions towards one or the other interpretation are short-lasting and unreliable [36–38]. Thus, perception alternates repeatedly between these interpretations although the sensory input stays unchanged.

Several authors interpret this endogenous perceptual change dynamics in the absence of an actual change in the sensory input as reflecting the fundamental rhythm of an inference process in the brain [20,22,39,40]. Particularly, the median “dwell times”, i.e. the durations of a temporarily stable percept during observation of an ambiguous figure, have been investigated in studies as operationalization for the duration of the ‘present moment’ regarding basic states of consciousness [23,41]. Dwell times during observation of the Necker cube have been analyzed in studies with experienced meditators in probing for trait-like changes in the perceptual dynamics pertaining to the present moment [42]. Practitioners of mindfulness meditation during actual meditation direct their attention continuously towards momentary bodily experiences, i.e. the breathing or while doing a mental body scan. As a consequence of this practice meditators acquire more efficient attention regulation capacities and memory formation is enhanced [43] (for a review of studies, see [44]). These trait-like effects come together with a longer duration of a present moment [23,41] and a subjectively felt slowing down of time in daily life [45]. Important in this context, experienced meditators are more successful in volitionally decreasing the reversal rate of ambiguous figures (thus increasing dwell times) as compared to non-meditating controls [28,40,42]. The decrease in reversal rates correlates with a latency increase of a P300-like event related potential (ERP) component, measured during a perceptual reversal of the Necker cube [40]. Based on the assumption that the mechanisms driving multistable perception reflect basic temporal constraints of consciousness states as described above, comparing them between experienced mindfulness meditators and non-meditating controls may help to elucidate the underlying mechanisms.

### EEG correlates of perceptual reversals during observation of ambiguous figures

The neural mechanisms underlying spontaneous perceptual reversals during observation of an ambiguous figure have been studied in a large number of psychophysiological studies (for reviews see [15, 46–48]). One basic problem for this line of research concerns the experimental access to the timing of a spontaneous perceptual reversal. It is particularly difficult to decide, whether potential psychophysiological markers precede a reversal and may thus be causal– or whether they are consequences of a reversal. The temporal resolution of fMRI is too low to answer this question [48]. EEG and MEG have in principle the necessary temporal resolution in the millisecond range. However, reaction times as reasonable time references for the reversal event, and therewith for the analysis of the physiological data, come with a considerable intra-individual temporal jitter of ± 100 ms [49,50] and are thus not precise enough.

**Fig 2:**
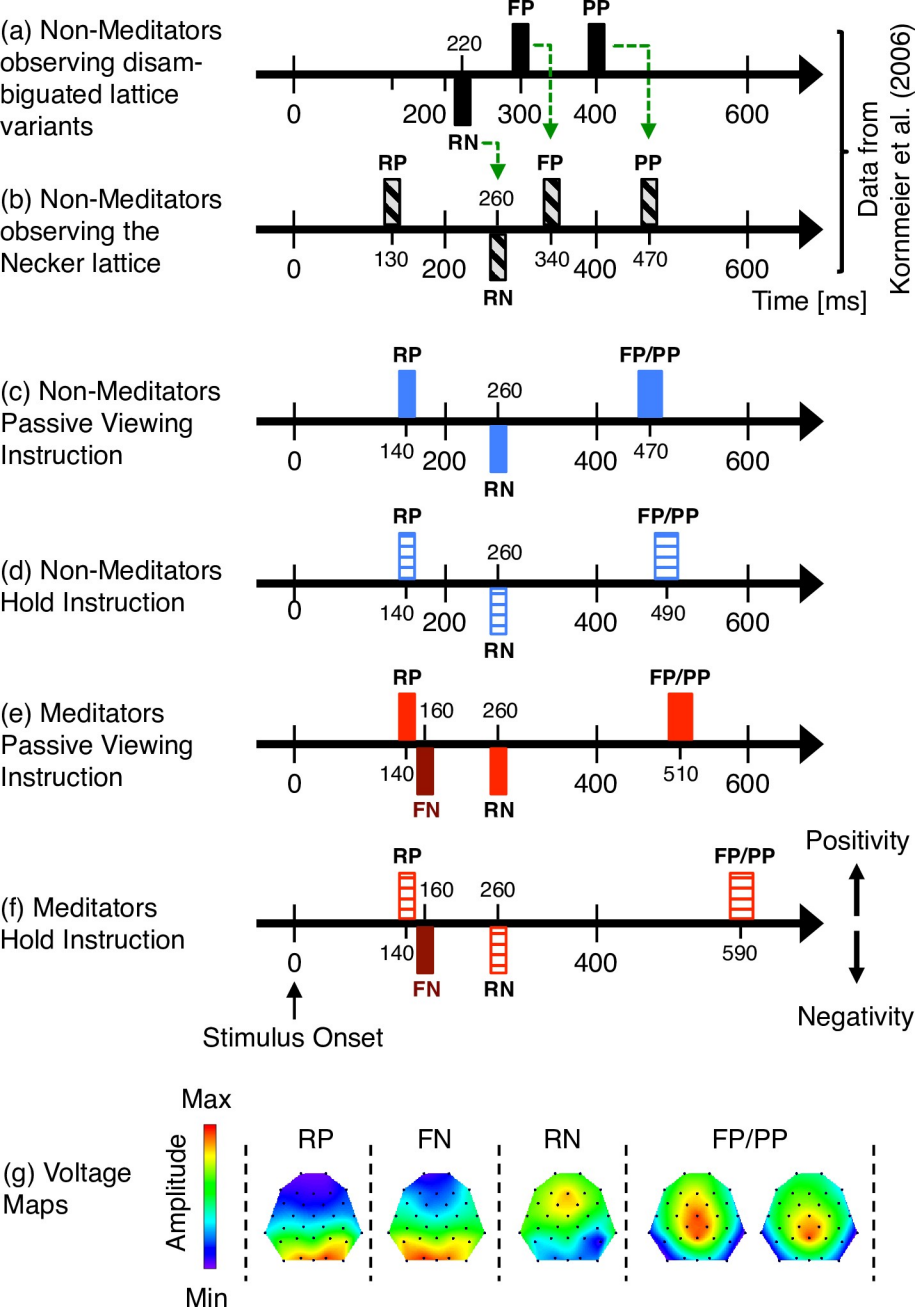
dERP chains and Voltage maps. Symbolic representations of the chain of dERP (difference ERP) components separately for non-meditators (blue and black) and for meditators (red). (a,b) dERP chain of non-meditators from Kornmeier et al. [59] for comparison. Green arrows: temporal delay between (a) exogenously induced and (b) endogenous reversals (= disambiguation time, see text). Data from Exp. 1 in (c,e) and data from Exp. 2 in (d,f). Both the Reversal Positivity (RP) and the Reversal Negativity (RN) show the same latencies across groups, experiments and studies. The Frontal Negativity (FN, dark red) occurs only in meditators. It is temporally close to the RP, but has an anterior distribution. The latency of the Parietal Positivity (PP) shows an increase from passive viewing (c,e) to hold instructions (d,f), and a tendency for longer latencies in meditators (e,f) compared to non-meditators (c,d). (g) Voltage map data of RP, RN and FP/PP are averaged across observer groups and experiments. Data for the FN voltage map are from meditators. RP: Reversal Positivity; FN: Frontal Negativity; RN: Reversal Negativity; FP/PP: Frontal/Parietal Positivity.

One solution to this time-reference problem is the so-called Onset-Paradigm. The initial ideas go back to psychophysical studies by Orbach et al. (e.g. [51]) and one EEG study by O’Donnell et al. [52]. Kornmeier and colleagues [50,53] have optimized the paradigm to the version also used in the present study. The basic idea is to present ambiguous stimuli discontinuously for about one second with each stimulus followed by a dark screen of 400 ms duration (defining an inter stimulus interval (ISI) between presentations). Participants compare their percept of the present stimulus with the preceding one and indicate in separate experimental blocks either perceptual reversals or perceptual stability (same percepts across two presentations) by key presses during the ISIs. This paradigm controls for low-level stimulus features (which should be identical in reversal and stability trials, simply because the same stimuli occur) as well as for neural activity related to the motor preparation and responses (most of which is postponed to the ISI). Most importantly the discontinuous stimulus presentation synchronizes the reversal events temporally with stimulus onset at a precision of ± 30 ms [54]. As a consequence, any EEG differences between the reversal and stability trials can be related to the perceptual reversal event. Consequently the ERP traces related to the stability trials were subtracted from ERP traces related to reversal trials and the difference ERPs (“dERPs”) were analysed with respect to deviations from zero. This Onset-Paradigm has so far been applied in several labs around the world and the following main findings have been substantially replicated ([55], for reviews see [47,56]):

Spontaneous perceptual reversals of the Necker cube are correlated with a chain of dERPs starting with an occipital positivity at 130 ms after stimulus onset (“Reversal Positivity, RP”), followed by an occipital/parietal Reversal Negativity (RN) at around 260 ms, a Frontopolar Positivity at 340 ms, and a Parietal Positivity 470 ms after stimulus onset (see Fig 2B). All dERPs, except the Reversal Positivity, are also present with exogenously induced perceptual reversals using two disambiguated variants corresponding to the two Necker cube percepts (see Fig 1B). However, they occur 40 to 70 ms earlier for exogenous (disambiguated stimulus variants) compared to endogenous reversals (ambiguous stimuli; compare Fig 2A and 2B).

According to the current interpretation of these results the conflicting visual information, provided by the ambiguous stimulus, is “recognized” in early visual areas not later than about 130 ms after stimulus onset (peak of the Reversal Positivity). The perceptual system then needs 40 to 60 ms to disambiguate the sensory information and to make a perceptual decision (average dERP latency differences between endogenous and exogenous reversals; compare Fig 2A and 2B). At the latest after 260 ms (peak of the Reversal Negativity, as the first dERP component occurring with both exogenous and endogenous reversals) the ambiguity has been resolved and the “normal” perceptual processing steps (as reflected by the exogenously induced reversals of the disambiguated stimulus variants) take place. The latency of the parietal positivity (between 400 and 470 ms) finally provides a temporal estimation of perceptual awareness of the reversal event. In the case of the ambiguous stimuli this takes place about 340 ms after the stimulus ambiguity has been recognized in early vision (with the ambiguous Necker cube) and 100–150 ms before participants’ manual responses.

Previous studies have demonstrated altered perceptions of Necker cubes in experienced meditators compared to non-meditating controls, probably indicating basic changes in endogenous brain dynamics. In the present EEG study we applied the Onset-Paradigm with Necker cubes as ambiguous stimuli to compare dERP correlates of the reversal dynamics in experienced meditators and non-meditating controls in an experiment with passive viewing instruction. In a second experiment participants tried to hold their current percepts volitionally as long as possible (hold condition). We used timings and locations of the individual components from the above-described reversal-related dERP chain as spatial and temporal regions of interest (“ROIs”) for our dERP analyses. Any difference along this chain between meditators and non-meditators may inform us precisely at which temporal processing step and roughly in which brain area meditation as long-term experience latest starts to fundamentally affect the neural systems.

## Methods

### Participants

17 experienced meditators (mean age 39 ± 8.4, 11 females, 15 right-handers, 2 ambidexters) took part in our study. We focused on meditation types with a dominant orientation toward awareness of the present moment (mindfulness meditation, Vipassana meditation, Soto Zen). Meditators had at least 3 years of continuous practice and had practiced at least 2 h per week over the last 8 weeks. The control group was matched for age and gender and consisted of 17 non-meditators (mean age 39.1 ± 7.9, 11 females, 14 right-handed). We had to exclude data from five meditators and two non-meditators because of too few perceptual reversals and thus too few EEG trials, resulting in 12 meditators (39.3 ± 9.5; 9 females, 11 right-handers, one ambidexter) and 15 non-meditators (38.2 ± 8; 10 females, 14 right-handed).

All participants were naive with respect to the specific experimental question and gave written informed consent. They had a visual acuity above 0.75, with one exception of a meditator with a visual acuity = 0.68, as measured by the Freiburg Visual Acuity Test (FrACT, [57]). The study was approved by the local ethics board (Ethikkommission der Universität Freiburg) and was performed in accordance with the ethical standards laid down in the Declaration of Helsinki [58].

### Stimuli

We used perceptually ambiguous Necker lattices as stimuli (Fig 1A). A Necker lattice is a combination of nine Necker cubes [1,50,53]. Size of the lattices was 7.51° × 7.51° visual angle and luminance was 173 cd/m^2^. A small cross in the center of the screen served as fixation target. All stimuli were generated with a Mac mini computer and presented on a Philips GD 402 monochrome monitor with a frame rate of 85 Hz.

### Procedure

We applied the Onset-Paradigm, as introduced by Kornmeier et al. [50,53]. Stimuli were presented discontinuously with 800 ms presentation time (± a random value between 12 ms and 100 ms) followed by short inter-stimulus intervals (ISI) of 400 ms. The study consisted of two experiments with two conditions each, conducted in one session. Each experiment consisted of a “reversal condition” and a “stability condition”. In the “reversal condition” participants compared successive stimuli and indicated perceived reversals of the 3D-lattice orientation from a from-above-perspective (Fig 1B left) to a from-below-perspective (Fig 1B right) and vice versa by pressing different keys on a keyboard (go/no-go task). In summary, if, in the reversal condition, participants perceive the current Necker stimulus in a perspective from above and have perceived the preceding stimulus in a perspective from below (or vice versa) they have experienced a perceptual reversal and indicate this by pressing the appropriate key.

In the “stability condition” participants indicated when they perceived the Necker cube unchanged in its 3D orientation from one stimulus to the next (separate keys for the two different lattice perspectives). Thus, if in the stability condition participants see the current stimulus in a perspective from above and have seen the preceding stimulus in the same way (or vice versa), they have experienced perceptual stability and only then press the appropriate key.

**Fig 3:**
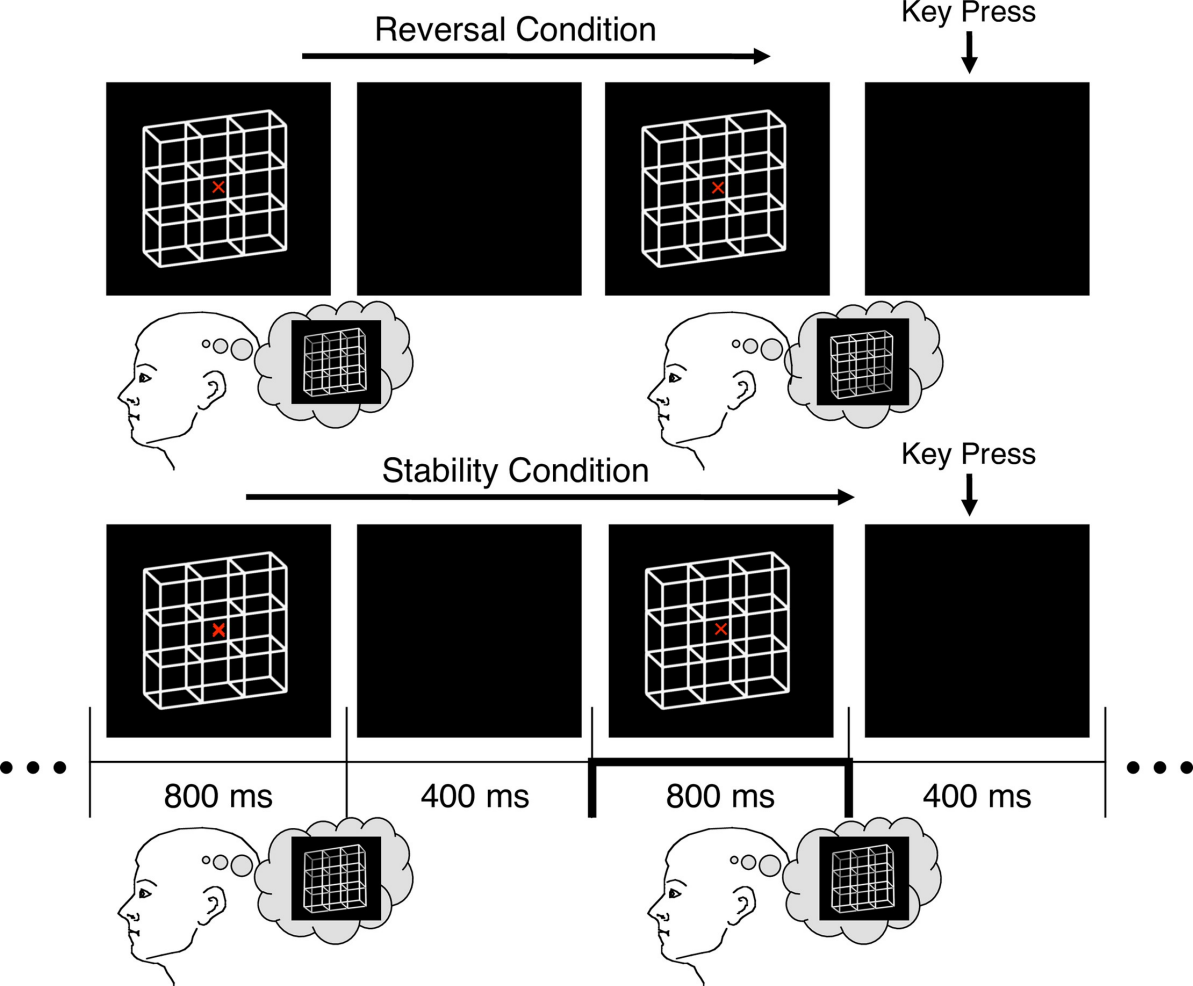
Onset-Paradigm. Ambiguous lattices were presented discontinuously with short blank screen ISIs in between. Participants compared successive stimuli and indicated in separate experimental conditions either perceptual reversals (Reversal Condition) or perceptual stability (identical percepts across presentations, Stability Condition) by key press in the 400 ms ISI (go-nogo task). We compared the reversal and stability ERP traces to the stimuli before the participants’ responses (indicated by the thick lines on the time axis).

Key presses had to be executed in the ISIs between the stimulus presentations in order to disentangle perception (reversal/stability) and motor related potentials (see Fig 3 for an overview). Any key press prolonged the subsequent ISI by 1000 ms as a temporal marker for a perceptual reset of participants. This was done because participants were instructed to restart the comparison task after the prolonged ISI and thus not to compare a stimulus immediately before with the stimulus immediately after key press and the prolonged ISI.

Experiments 1 and 2 were identical with one exception. In Experiment 1 participants had to keep a passive attitude towards the stimulus without provoking or inhibiting perceptual reversals. In Experiment 2 participants were instructed to mentally prevent reversals and instead try to hold the current percept as long as possible.

Each experimental condition (reversal and stability) was repeated once, resulting in a total of 8 experimental blocks (2 experiments x 2 conditions x 2 realizations). The two experiments were executed in one session, which lasted for about 1.5 hours.

Participants’ mental effort to hold one percept of the ambiguous Necker lattice may disable the ability to adopt a passive attitude towards the same stimulus in a subsequent experimental block. In order to avoid this, Experiment 1 (passive viewing instruction) took place in the first half of the session and Experiment 2 (hold instruction) in the second half. The order of conditions (reversal and stability) was mirror-symmetric within and randomized between participants.

### EEG recording

EEG signals were measured from 32 active electrodes (extended 10–20 system, [60]) using the Brain Vision EEG system (Brain Products GmbH, Gilching, Germany) with the central midline electrode as online reference. Impedance was kept below 15 kΩ for all electrodes. The EEG signals were amplified with a factor of 1000, band pass filtered at 0.1 to 100 Hz, digitized with a sampling rate of 500 Hz and streamed to disc. We re-referenced the EEG data offline to the average of electrodes TP9 and TP10, and removed EEG trials containing amplitude excursions exceeding ±150 μV.

### Data analysis

We sorted the EEG trials where participants had responded (go trials) with respect to experiment (passive and hold instructions), condition (reversal, stability), and EEG electrode, and averaged them selectively to ERPs. ERP computation included digital filtering with a latency-neutral low-pass filter with a cut-off at 25 Hz. For baseline correction we used the average amplitude from 60 ms before to 40 ms after stimulus onset. The two experimental conditions, reversal and stability, served for data analysis purposes: ERPs of the stability conditions were subtracted from ERPs of the reversal condition in order to remove stimulus-related low-level signals, which were assumed to be identical in both conditions, and to isolate components related to the endogenous perceptual reversals of the Necker lattice in the resulting difference ERP traces (“dERPs”). Table 1 reports statistics about the number of trials entering the data analysis.

**Table 1:** Trial number statistics.

Experiment / Condition	Average total trial number (± SD)	Total trial number after artefact rejection	Maximum trial number	Minimum trial number
**Non-Meditators Passive Viewing**	78.7 (± 26.2)	70.4 (± 21.6)	105	34
**Non-Meditators** **Hold-Instruction**	61 (± 28.4)	57.7 (± 27.1)	95	26
**Meditators** **Passive Viewing**	65.8 (± 34.6)	58.8 (± 34.4)	95	12
**Meditators** **Hold-Instruction**	43.3 (± 25.4)	42.2 (± 24.8)	95	13

We performed two types of analyses:

In the first hypothesis-driven analysis we focused on three spatio-temporal regions of interest (ROI) for the reversal-related ERP components as described in previous studies (e.g. [50,59], for a review see [47]). Peaks were automatically identified as the largest local maximum/minimum in the given temporal ROI relative to the baseline. In the case of no local maximum/minimum, the algorithm averaged the amplitudes across the whole ROI.

The first ROI was related to the Reversal Positivity and was restricted to the occipital and parietal electrodes O1, Oz, O2, P3, Pz and P4 and to a time window from 80 ms to 250 ms after stimulus onset. Within this ROI we identified amplitude and latency of the maximal positive deflection from the dERPs.

The second ROI was related to the Reversal Negativity and was restricted to the electrodes O1, Oz, O2, P3, Pz and P4 and to a time window from 150 ms to 350 ms after stimulus onset. Within this ROI we identified amplitude and latency of the maximal negative deflection from the dERPs relative to the baseline.

The third ROI was related to the Parietal and Frontopolar Positivities and was restricted to the electrodes Pz, Cz and Fpz and to a time window from 250 ms to 700 ms after stimulus onset. Within this ROI we identified amplitude and latency of the maximal positive deflection from the dERPs relative to the baseline.

A summary of spatial and temporal ROIs for the three ERP components can be found in Table 2.

**Table 2:** Overview of hypothesis-driven analyses.

dERP Component	Temporal ROI	Spatial ROI
Reversal Positivity	80–250 ms	O1, Oz, O2, P3, Pz, P4
Reversal Negativity	150–350 ms	O1, Oz, O2, P3, Pz, P4
Parietal/Frontopolar Positivity	250–700 ms	Pz, Cz, Fpz

We tested for the presence of each of the three reversal-related dERP components by calculating running t-tests, where the difference trace was tested against zero within the respective temporal ROIs (e.g. [61]), separately for each electrode from the spatial ROIs and separately for each group and experimental condition. The respective component was regarded as present if the p-values (uncorrected) of at least 10 successive data points (i.e. 20 ms) within the respective temporal ROI were smaller than 0.01 from at least one electrode from the spatial ROI. This criterion is admittedly liberal and potentially prone to type 1 errors, in particular because we did not correct for multiple testing. However, we regard it still as defensible by two reasons: First, the principle proof of the existence of the three reversal-related dERP components comes from replications of these findings from other labs around the world (e.g. [55], for reviews see [47, 56]). Second, it is well known that both the Reversal Positivity and the Reversal Negativity are small effects with small effect sizes [47]. More conservative criteria would thus have increased the beta error probability.

All three components (Reversal Positivity, Reversal Negativity, Parietal Positivity) fulfilled this criterion.

For each of the three components we then calculated separate ANOVAs with the between-subject factor GROUP (meditators and non-meditators) and the within-subject factors EXPERIMENT (passive, hold) and ELECTRODE (depending on the analysed components and spatial ROIs), and amplitude and latency of the respective component as dependent variables.

In an additional exploratory analysis we looked for further deflections in the grand mean dERP traces outside the spatio-temporal ROIs with significant deviations from zero with a p-value (running t-test) below a pre-defined alpha value of 0.01 for at least 10 data points (i.e. 20 ms) in at least two neighboring electrodes. We found an anterior negative deflection at around 160 ms after stimulus onset in the dERPs of the meditator group but not in the control group (see results). We assessed individual negative peaks of this deflection, focusing on a spatial ROI encompassing central (C3, Cz, C4), frontal (F3, Fz, F4) and frontopolar (Fp1, Fp2) electrodes, and on a temporal ROI from 80 ms to 250 ms after stimulus onset. These data were entered into a subsequent ANOVA with the between-subjects factor GROUP (meditators and non-meditators) and the within-subjects factors EXPERIMENT (passive, hold) and ELECTRODE, and with amplitude and latency as dependent variables.

### Source reconstruction

The sources of the above described dERP components have been already calculated elsewhere [62,63]. In our exploratory analysis we identified a novel dERP component, which was only found in meditators (see Results section). In order to collect more information about this meditator-specific dERP signature, we estimated the underlying neural sources, using MATLAB 2018b (The MathWorks, Inc., Natick, Massachusetts, United States) and EEGLAB v.14.1.2 [64]. Sources were reconstructed using the Source App [65], an EEGLAB extension for testing various Forward Models and Inverse Solutions provided by Fieldtrip [66] and SPM12 (revision 7487, https://www.fil.ion.ucl.ac.uk/spm/software/spm12/).

Reliability of results can be increased by combining EEG measurements with fMRI T1 scans and taking into account individual brain anatomy data. No T1 data were available for the present analysis. Therefore the present results need to be taken with caution, given the inverse problem with EEG data.

In order to reduce spatial (and temporal) inaccuracies, a multitude of algorithms for MEG/EEG source analysis were introduced in the past decades, each of which has its own advantages and disadvantages. Minimum norm based inverse solutions, such as exact low resolution tomography (eLORETA), can yield remarkable accuracy for single simulated dipoles even in the presence of structured noise [67]. Simulations, however, showed that it can perform rather poorly when multiple sources are active. Another commonly used family of inverse solutions are beamforming techniques, e.g. linear constraint maximum variance (“LCMV”, [68]), which can lead to higher localization accuracies. LCMV beamforming, however, can be disturbed by correlated source activity, which then leads to the suppression of sources.

For the purpose of the present analysis we decided for an algorithm that is capable of identifying multiple active and possibly correlated sources, the multiple sparse priors algorithm (“MSP”, [69]), which concurrently compensates for both problems mentioned above and also produces smaller localization errors than LORETA. Sources were estimated using a canonical cortical mesh as source model, a three-shell boundary element method (“BEM”) head model and MSP using Greedy Search. All conditions and subjects were inverted simultaneously using group inversion [70]. Thus, the same sources were assumed for each participant while time courses can differ. This was shown to further enhance localization accuracy. The number of sparse priors was set to 64 and smoothness of source priors was 0.6 mm.

### Source statistics

After group inversion sources were exported from SPM12 to the Fieldtrip structure and contrasted at the time range of interest. One-sample t-tests were performed for each vertex of the cortical mesh. The resulting statistical image was then thresholded at a predefined α = 0.05, and source activations were grouped using 1000 repetitions of k-means clustering and the elbow method (cutoff: 0.95, max. number of clusters per hemisphere: 8) for determining the optimal number of clusters. Anatomic regions were labeled using the automatic anatomical labeling (AAL) of activations (125).

## Results

**Fig 4:**
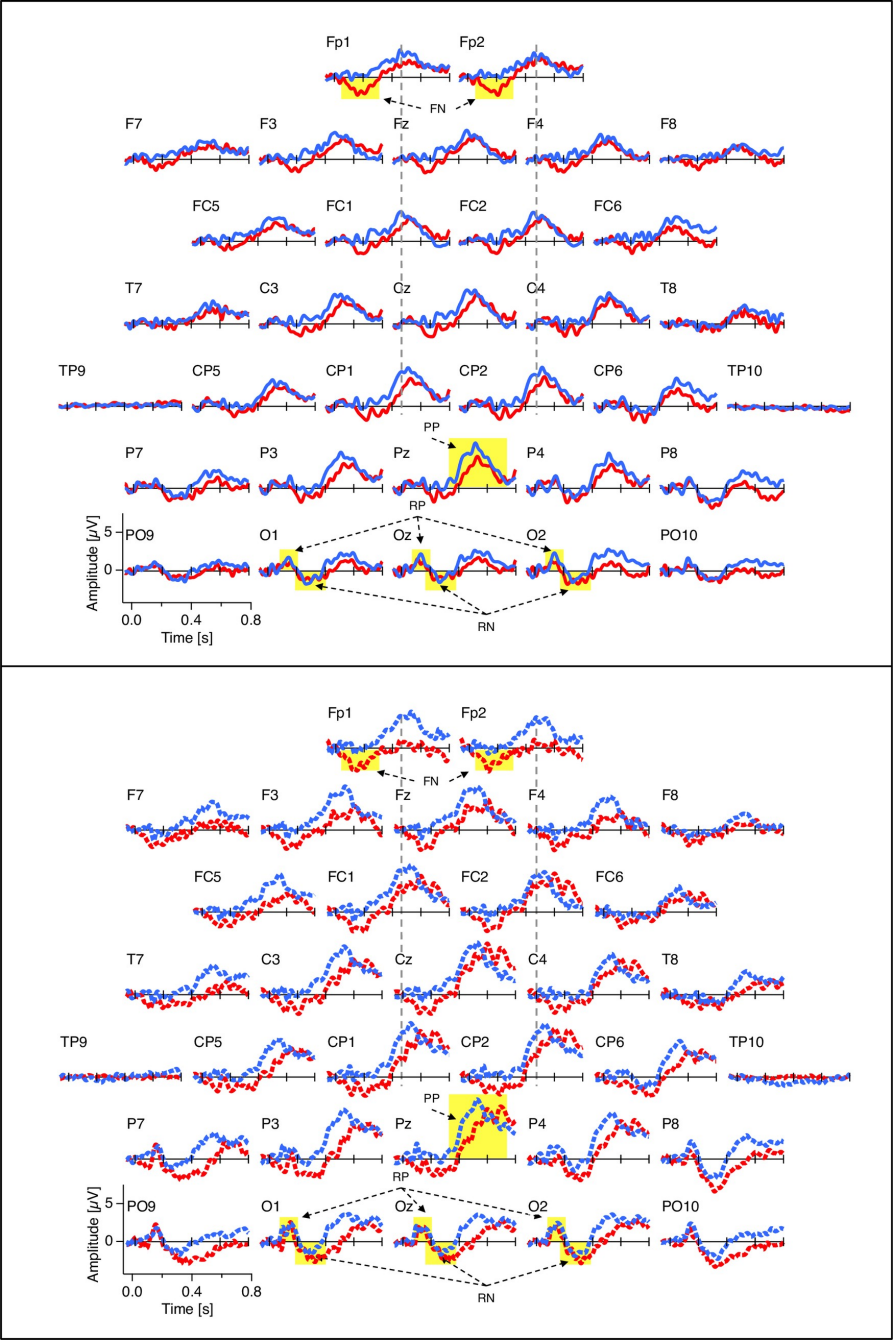
dERP traces. Grand mean difference ERP traces (dERP; reversal minus stability) at each of the 32 electrode positions. Top: Experiment 1 with passive viewing instruction. Bottom: Experiment 2 with hold instruction. Red traces: meditators; blue traces: non-meditators. Yellow areas indicate the analysed dERP components. RP: Reversal Positivity; RN: Reversal Negativity; PP: Parietal Positivity; FN: Frontal Negativity (only visible in meditators).

**Fig 5:**
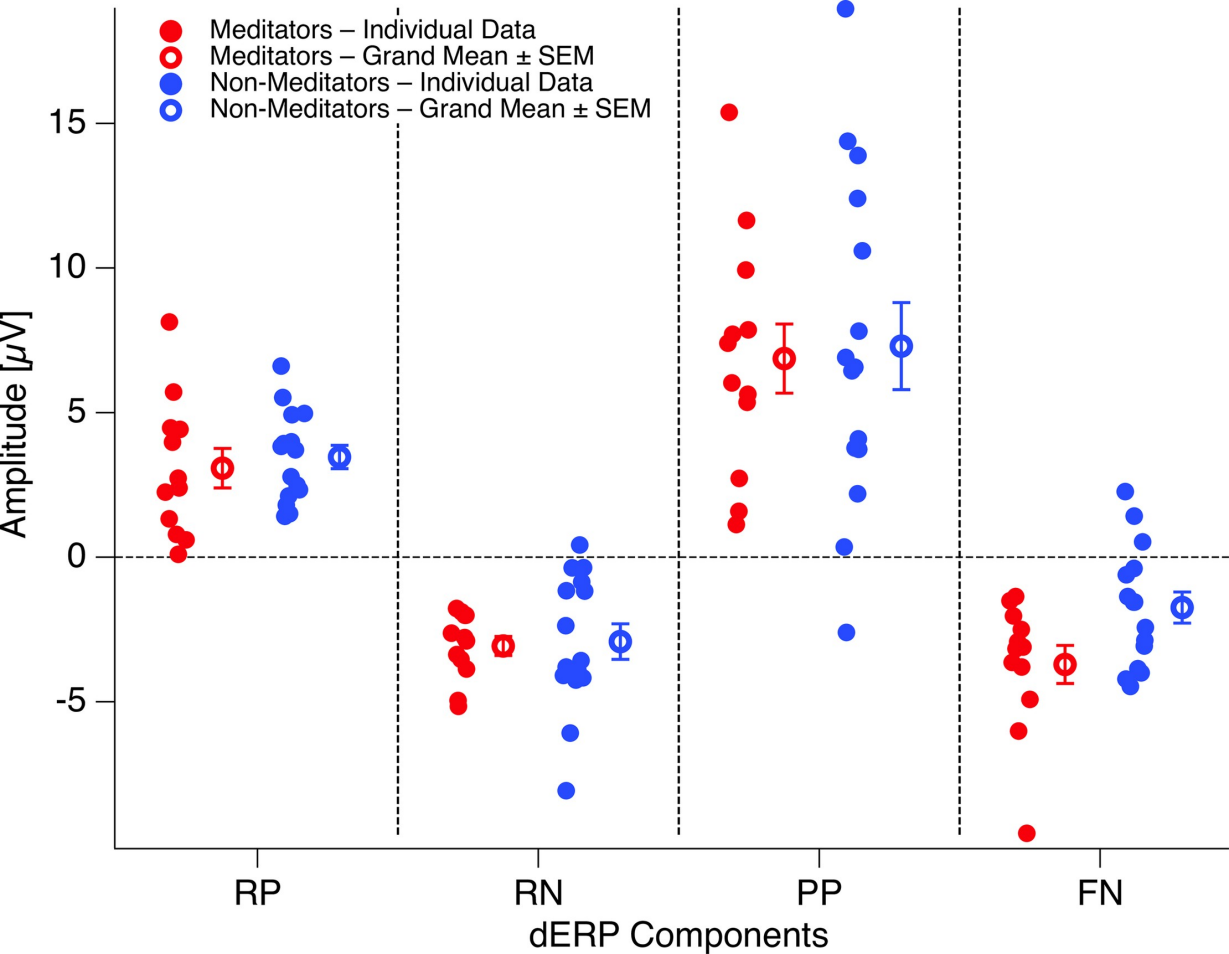
Individual dERPs. Filled circles represent the individual peak data, averaged across Experiments 1 and 2 for the different dERP components analysed. Open circles and antennas indicate grand means ± SEMs. Red: meditators; blue: non-meditators. For the vast majority of participants and dERP components the amplitude values are systematically above (RP and PP) or below (RN, FN) zero, with exception of the amplitude values in the FN time window for non-meditators. RP: Reversal Positivity; RN: Reversal Negativity; PP: Parietal Positivity; FN: Frontal Negativity.

In the following we report the dERP effects within our predefined spatio-temporal ROIs. Fig 4 depicts the grand mean dERP traces, separately for each EEG electrode. Fig 5 presents the individual data for each reported dERP component. Finally, Fig 2(G) provides voltage maps to each dERP component and Fig (2C–2F) provides a schematic overview of the temporal succession of the dERP components, separately for each group of participants (meditators and non-meditators) and for the two Experiments (passive viewing and hold instruction). The time line of the current results can be compared with the time line of a previous study using the same experimental paradigm (Fig 2A and 2B).

### Reversal positivity

The ANOVA indicated no difference between meditators and non-meditators for the variable amplitude, no difference between experiments (passive viewing vs. hold), nor any interaction for the Reversal Positivity. The ANOVA indicated a significant difference between electrodes (p = 0.0002; F(5,125) = 5.26, *η*^*2*^_*p*_ = 0.18).

Post-hoc Wilcoxon tests indicated larger amplitudes of the Reversal Positivity at occipital compared to parietal electrodes (p = 0.01) and a slight lateralization to the right (p = 0.01; see also voltage map in Fig 2G). None of the post-hoc results survived the Bonferroni-Holm correction for multiple testing [71].

We found no significant effect for the variable latency.

### Reversal negativity

The ANOVA indicated no significant effect and no significant interaction for the variable amplitude. For the variable latency we found a significant effect for the factor electrode (p = 3.5 x 10^−13^, F(8,200) = 11.31, *η*^*2*^_*p*_ = 0.34) with shorter latencies at occipital compared to parietal electrodes (corrected/uncorrected post-hoc p-values: p = 0.02/0.002).

### Parietal positivity

For the variable amplitude, the ANOVA indicated significant effects for the factors experiment (p = 0.03, F(1,25) = 5.22, *η*^*2*^_*p*_ = 0.33) and electrode (p = 1.6*10^−7^, F(7,175) = 7.16, *η*^*2*^_*p*_ = 0.44). The post-hoc tests indicate significantly larger amplitudes at central compared to peripheral electrodes (corrected/uncorrected p-values: p = 0.0036/0.0003). For the variable latency, the ANOVA indicated significant effects for the factors experiment (p = 0.042, F(1,25) = 4.6, *η*^*2*^_*p*_ = 0.15) and electrode (p = 3.8*10^−8^, F(7,175) = 7.74, *η*^*2*^_*p*_ = 0.23) and a trend torwards longer latencies in meditators compared to non-meditators (p = 0.054, F(1,25) = 4.1, *η*^*2*^_*p*_ = 0.24). The post-hoc tests indicate significantly longer latencies at parietal compared to central electrodes (corrected/uncorrected p-values: p = 0.0023/0.023) and compared to the frontopolar electrodes (corrected/uncorrected p-values: p = 0.032/0.0036).

The dERP traces from both experiments are depicted separately for meditators and non-meditators in Fig 4. Individual and grand mean peak data are presented in Fig 5. A schematic display of the chain of dERP components related to reversals of the ambiguous Necker lattices can be seen in Fig 2.

Overall we largely replicated the chain of dERP components related to endogenous perceptual reversals of the Necker lattice. Remarkably the individual components of this chain are highly similar between experiments (passive viewing and hold instructions) and between experimental groups (meditators and non-meditators). The only difference between experiments and groups (only visible as a tendency) is related to the latency of the Parietal Positivity. The latencies of the Parietal Positivity are higher in Experiment 1 (hold instruction) than in Experiment 2 (passive viewing instruction). Further, there is a tendency for increased latencies in meditators as compared to non-meditating controls (p at about 0.05). Notice that the present analyses did not focus on behavioral results (reversal rates/dwell times). A focused latency analysis of the Parietal Positivity together with an analysis of the effects of meditation on reversal rates have been published elsewhere in the context of the formal Necker Zeno Model of bistable perception [40]. There we found a weak but significant latency difference (p = 0.02) between groups, when focusing only on parietal electrodes. We further replicated the generally lower reversal rates if participants tried to hold their percept than if they observed it with a passive attitude. We further replicated the finding of lower reversal rates and more volitional control over the perceptual dynamics in meditators compared to the non-meditators.

### Frontal negativity

As shown in Fig 4, meditators showed an early (160 ms after stimulus-onset) reversal-related Frontal Negativity (FN) that was absent in non-meditators, as indicated by the ANOVA for the variable amplitude and the factor Group (p = 0.013, F(1,25) = 7.2, *η*^*2*^_*p*_ = 0.68). None of the other factors and no interaction were indicated as significant.

**Fig 6:**
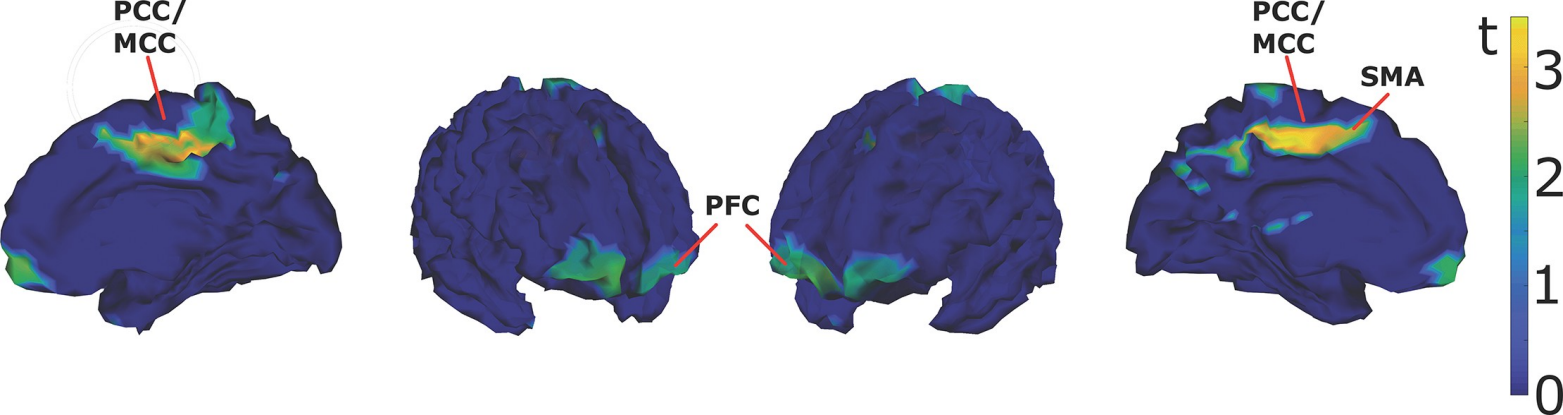
FN sources. Sources of the Frontal Negativity (reversal vs. stable perception) in Meditators. In order to make the calculated sources easier visible we used in these graphs an uncorrected threshold of p = 0.1 for the selection of t-values. PCC: Posterior Cingulate Cortex; MCC: Middle Cingulate Cortex; PFC: Prefrontal Cortex; SMA: Supplementary Motor Area.

To investigate possible neural generators of the FN, reversal trials were contrasted against stability trials. The calculated sources were first averaged across a broad time window from 80 to 250 ms before calculating the t-statistic for each pair of voxels. Statistical images, indicating t-values of the source activity of the Frontal Negativity in meditators are depicted in Fig 6 and an overview of the significantly activated clusters is provided in Table 3.

**Table 3:** FN sources.

Position	Anatomic Label	T	*p*
-9–21 48	Cingulum_Mid_L	3.08	0.013
-9 1 53	Supp_Motor_Area_L	2.52	0.015
-11–5 51	Cingulum_Mid_L	2.6	0.035
17–29 42	Cingulum_Mid_R	3.46	0.035
-11–5 51	Cingulum_Mid_L	2.6	0.035
-5–16 49	Supp_Motor_Area_L	3	0.037
6 52–14	Frontal_Med_Orb_R	2.34	0.037
-6–33 51	Paracentral_Lobule_L	3.12	0.040
8–12 50	Cingulum_Mid_R	3.46	0.040
-14–40 53	Cingulum_Mid_L	2.97	0.044

Statistically significant clusters (reversal vs stability) related to the Frontal Negativity (FN) in meditators together with t-values and p-values (uncorrected). L: left hemisphere; R: right hemisphere.

Source analysis of the Frontal Negativity using MSP reveals two major regions that contribute to the dERP. Meditators show increased activity in middle/posterior cingulate cortex (now referred to as PCC) and left supplementary motor area (SMA). In more frontal sites, right medial prefrontal cortex (mPFC) showed increased reversal-related activity.

## Discussion

For both meditators and non-meditators we found highly similar chains of dERP components, related to spontaneous perceptual reversals of discontinuously presented ambiguous Necker lattice stimuli. These dERP chains started with a posterior Reversal Positivity with maximum amplitude at occipital electrodes and a peak latency of around 140 ms after stimulus onset. The Reversal Positivity was followed by a posterior Reversal Negativity with maximal amplitude at occipital and parietal electrodes and a peak latency of around 260 ms after stimulus onset, and finally by a large and broad positive dERP excursion, extending from parietal to frontopolar electrodes with a maximum at midline parietal and central electrodes and a varying peak latency of around 480 ms at frontal and frontopolar electrodes, and around 540 ms at parietal electrodes (see dERP traces in Fig 4, individual data in Fig 5 and a schematic display in Fig 2). These findings largely replicate previous results about both spatial and temporal features of dERP correlates of perceptual reversals ([53,72,73,50,54,74,75,63,76–78,55,79,8,80], for reviews see [56,47]).

In addition to the common findings across the two groups and experiments (passive viewing, hold instruction) we also found a difference between experienced meditators and non-meditating controls: An early (around 160 ms after stimulus onset) Frontal Negativity with maximal excursions at anterior electrodes was identified in experienced meditators but not in the non-meditators.

### Possible limitations of the present findings

Before we interpret these findings, we discuss potential limitations to our approach.

#### Perceptual reversals and presentation mode

One basic ingredient of the Onset-Paradigm is the discontinuous presentation of the Necker lattice stimulus with short blank-screen interruptions between stimulus presentations. An important question is, whether perceptual reversals during such interrupted observations are a good model for reversals during continuous stimulus observations? Or in other words: Is the change to a different 3D percept of the lattice after the blank screen interval a *reversal* or rather a *novel percept* and thus unrelated to the previous one, as e.g. argued by Noest et al. [81] or Kleinschmidt et al. [15]?

The following evidence favors the validity of our approach:

(1) No Vision without Interruption: Typical eye-blinks last for about 200 ms, occur every 4 s on average (e.g., Caffier et al., 2003) and, importantly, they interrupt the continuous visual input. Thus, even during continuous stimulus presentations visual input is repeatedly interrupted.

(2) Confirmatory evidence from studies with interrupted presentations of ambiguous figures: Several studies, presenting ambiguous figures discontinuously, found huge variations of the reversal rates (number of reversals per time unit) as a function of gap durations (e.g. [51,72,82–84]). Importantly, this function is nonlinear with a smooth monotonous increase of reversal rates from continuous to interrupted presentations up to gap durations of about 400 ms and monotonously decreasing reversal rates up to zero reversals with longer gap durations (see also Fig 3 in [47] for a graphical representation of this relation). This non-linearity may indicate a threshold between *reversals* (gaps up to 400 ms) and *novel percepts* (gaps larger than 400 ms in [47]; see [85,86,87] for a more detailed discussion of this issue).

(3) ERP comparison with continuous presentation of ambiguous figures: We replicated findings of a P3b-like parietal/central ERP positivity reported in studies with continuous presentations of the Necker cube, where reaction times were taken as time reference for averaging (e.g. [49,88,89]). The gain in temporal resolution with our paradigm allowed us the identification of additional, narrower, and smaller components beyond the broad P3b-like component, which are less robust concerning the temporal jitter of at least ± 100 ms [50] between EEG trials introduced by reaction time variability when reaction times served as time reference for averaging (for more details see [47]).

#### Exploratory analysis and type I errors

In addition to the hypothesis-driven analysis we conducted an exploratory analysis in order to compare the dERP data between meditators and non-meditators. We had no *a priori* hypotheses concerning time period and brain area of potential differences in the perceptual processing of the two groups and thus looked at all EEG electrodes and the whole stimulus presentation time window of 800 ms (see Methods section). For this analysis we defined a moderate alpha threshold of 0.01 but did not systematically correct for multiple testing, because our exploratory analyses had the aim to identify possibly meaningful patterns for confirmatory testing in future replication studies. Given this exploratory approach, the findings of the Frontal Negativity, exclusively in the meditators group has to be taken with caution.

### What happens in the brain when perception changes but not the stimulus?

Postulating that reversals during our discontinuous stimulus presentation with short gaps provide an adequate model for the continuous case, how can the chain of ERP components be interpreted?

In their review from [47], Kornmeier & Bach describe a perceptual reversal as consisting of two separate processes taking place on different time scales.

#### Destabilisation

During observation of ambiguous figures, perception is temporarily stable between two reversals. The neural representations underlying such transiently stable percepts slowly destabilize over time, probably due to lower-level adaptation processes (e.g. [51,81,90–94]). The durations of such transient perceptual stability periods are in the range of ± 4 s but vary considerably between observers (e.g. [15,22, 95–98]) and also between different types of ambiguous figures (e.g. [99]). Depending on the degree of stability of an observer’s percept at a certain moment in time and on the type of the observed ambiguous stimulus he/she observes, reversals occur earlier or later as a function of volition and selective attention [2–4,77,89,92,100–103], sensory transients [2,51,82,104,105], eye-movements [106–111] or other potentially not yet identified factors. Further, the pre-onset activity in brain areas specific to the respective perceptual interpretations can affect the perceptual outcome (e.g. [112]).

So far, there is no clear physiological signature available that can be unequivocally causally related to the slow destabilization process. Interestingly, both we [113,114] and Britz et al. [62], using both the above described Onset-Paradigm, found right-hemispheric neural activity in the temporal gap between two stimulus presentations involved in a perceptual reversal. This signature might reflect the “end of the destabilization process” and predict an upcoming reversal in the immediate future (i.e. with the next stimulus in the presentation sequence of the onset paradigm). It is in accordance with a number of other studies reporting right-hemispheric brain activity in the context of spontaneous perceptual reversals, but with a less precise temporal resolution of the neural processing (for reviews see [47,48]).

#### Disambiguation/Restabilisation

During a perceptual reversal at the transition from one stable perceptual state to the next, the brain of an observer enters a transient state of maximal instability. Kornmeier & Bach found an early positivity (Reversal Positivity, 130 ms after onset), which was restricted to spontaneous endogenous reversals of the ambiguous stimuli. No such component was found with exogenously induced (i.e. computer-generated) reversals of disambiguated stimulus variants (see Fig 2A and 2B). They interpret this Reversal Positivity as an index of a temporally slightly extended state of maximal perceptual instability during the processing of ambiguous visual information [47]. The Reversal Positivity is a very small deflection and has not been replicated in all studies using the Onset-Paradigm (e.g. [63]), probably due to the low signal-to-noise ratio. In the present study, we replicated the Reversal Positivity with both meditators and non-meditators and in both the passive and the hold conditions and found neither a difference between groups nor between experiments (see Fig 2). All the subsequent reversal-related ERP signatures, the Reversal Negativity and the Parietal and Frontopolar Positivities were reported to be present with both endogenous and exogenously induced reversals (e.g. [47]).

From an evolutionary perspective such states of perceptual instability may have been critical, because moments of uncertainty about the outside world reduce precision and delay latencies of (re-) actions and therewith increase the risk for the individual. Thus, most probably there may have been evolutionary pressure to minimize the durations of such unavoidable and at the same time critical unstable states in order to minimize uncertainty and the related risks. Minimizing the time of unstable perceptual states may become difficult in situations with maximal ambiguous sensory information, like during the observation of an ambiguous figure. In such situations the duration of the unavoidable unstable brain state may be slightly increased, as found in the EEG studies. According to Kornmeier & Bach [47], the relatively short temporal delay of the subsequent dERP components of at least 50 to 60 ms in the case of the endogenous reversals, compared to exogenously induced reversals indicates that this additional time is necessary to disambiguate the ambiguous sensory information (‘disambiguation time’, see green dashed arrows in Fig 2A and 2B). As a consequence, all the components following the Reversal Positivity occur after successful disambiguation and are thus secondary with respect to the reversal event. Interestingly, a number of imaging studies also report bilateral reversal-related activity in parietal and frontal/frontopolar areas (e.g. [15,48,115]). If the ERP signatures (Reversal Negativity and the subsequent Parietal and Frontal/Frontopolar Positivities) and the fMRI signatures reflect the same underlying process, one can speculate that those fMRI signals also reflect effects secondary to the reversal event (except the above reported right-hemispheric signatures).

In the present study we also replicated the dERP components subsequent to the Reversal Positivity, starting with the Reversal Negativity, which again shows no differences between experiment type (passive viewing, hold condition) nor between meditators and non-meditators. We further replicated the Parietal Positivity, with an increase in latency in the hold condition (across groups) and a tendency for general longer latencies in meditators compared to non-meditating controls.

Previous studies found a distinct Frontopolar Positivity preceding the Parietal Positivity by about 100 ms [47]. We found a tendency for shorter latencies at frontal and frontopolar electrodes, however with a much smaller latency difference of only about 6 ms compared to earlier studies.

The finding of longer latencies of the Parietal Positivity in the hold compared to the passive condition and the tendency for longer latencies in meditators compared to non-meditators are interesting in the context of the above described explanation: These latency effects correlate positively with dwell times effects. A more elaborate latency analysis of the Parietal Positivity together with a focused analysis of behavioral data (reversal rates / dwell times) from this study have been published elsewhere in the context of the formal Necker Zeno Model of bistable perception [40]. The model provides a simple mathematical relation between three time scales that have been often discussed in cognitive science. One time scale is represented by the dwell times and is discussed as a measure of the duration of a present moment [23,41]. Another time scale is represented by the latency of P300-like ERP components, like the present Parietal Positivity. These latencies have been discussed as measures of the time necessary to become aware of a sensory (visual) stimulus (e.g. [116]). Interestingly, the Necker Zeno Model postulates that unstable systems in cognition, like unstable systems in physics, should show the relation provided by the model and the data from the present study provide evidence, as elaborated in [40].

### What is different in the brains of meditators during perceptual reversals– and in general?

In the present study we focused on the processes underlying a perceptual reversal and its later cognitive registration. Analyses in the frequency domain related to processes preceding and potentially leading to perceptual instability (destabilization) and perhaps inducing a perceptual reversal are in preparation and will be presented in a separate paper elsewhere. Here, we focused on dERPs and replicated–for both meditators and non-meditating controls–the dERP correlates of what Kornmeier & Bach [47] conceptualized as a transient state of maximal perceptual instability (i.e. the Reversal Positivity) and a fast disambiguation process thereafter.

In meditators we identified an additional reversal-related early, Frontal Negativity, which did not differ between a passive viewing attitude (Exp. 1) and intentions to hold the current percept as long as possible (Exp. 2). Remarkably, this Frontal Negativity occurred at about the same early time as the Reversal Positivity (on average about 10 ms later, at about 160 ms after stimulus onset) and is thus most probably also related to the transient state of perceptual instability and/or the subsequent fast disambiguation/restabilization, as elaborated above. Further, it can be assumed to be a trait-like signature of long-term meditation proficiency, since the meditators were not in a meditative state during our experiments.

To the best of our knowledge, we are the first to report such a component, although there are also reports of other anterior EEG signatures related to meditation praxis (e.g. [117,118]). EEG source analyses identified mPFC and PCC as potential generators of this component. Interestingly, in the literature BOLD and EEG signal responses in the default mode network, encompassing mPFC and PCC, were found to be reduced in meditators during meditation as well as during performance of cognitive tasks. These reductions are interpreted as marker for increased attention and less mind wandering (e.g. [119–122]). The present Frontal Negativity is a differential ERP signature (dERP) and may reflect a difference in activation in these areas related to effortless (non-distracting) stability trials versus (distracting) reversal trials where attention may become less focused for some time. The Frontal Negativity may thus reflects some kind of meta-cognitive (or meta-perceptual, e.g. [123]) monitoring and/or control function.

In the following this tentative working hypothesis concerning the functional role of the Frontal Negativity will be worked out in more detail:

Any change in perception can be described as a transition from one perceptually stable brain state to another stable state. As already discussed above, evolutionary pressure may have optimized our perceptual system to minimize the duration of the inevitably unstable brain states at the transitions between stable states in order to reduce related uncertainty and danger as much as possible in a highly automatized manner. In our modern societies danger from predators is no longer omnipresent and energy (food) is abundantly available (at least in the industrial societies). Deceleration of perceptual construction and/or brain processing in general and the temporal extension of unstable brain states, sometimes also labelled as ambiguity tolerance (e.g. [124]), may still be dangerous in certain situations, e.g., during car driving, etc. However there are numerous uncritical situations where decelerated perception and ambiguity tolerance are safe and can be even advantageous. Recent theoretical approaches describe unstable mental states as ‘acategorial states’ and postulate that the temporal extensions of such acategorial states promote creativity and processes related to insight experiences. Further, they may be necessary preconditions of meditation-induced altered states of consciousness [19,40,125,126].

We postulate that acategorial mental states correlate with acategorial brain states. Long-term effects of extensive meditation practice may include the ability to interrupt and more generally control phylogenetically evolved automatisms in perceptual processing and beyond. As one consequence meditators are able to optimize attentional focus, thereby ignoring external distractors to a larger extend. It has indeed been shown that experienced meditators perform better in various attention and working-memory tasks [43,127] and have lower scores in self-reported impulsivity [45]. Experienced meditators may further be able to temporally extend acategorial mental states and the related acategorial brain states and/or to tolerate them. Extensive meditation training may be necessary to acquire these capabilities, because of the robustness of the perceptual automatisms. The identified source brain areas, like the mPFC and PCC, may be functionally relevant in this context.

Ambiguous figures are artificial stimuli, where two interpretations are about equally probable. Disambiguation of the sensory information may become more difficult during observation of an ambiguous figure and transient acategorial states of perceptual instability may thus become slightly longer (by about 50 ms [47]). While experienced meditators may have acquired the ability to induce such acategorial brain states volitionally, the related frontal brain may also be activated in cases when slightly prolonged acategorial brain states are induced by an ambiguous figure.

These temporally extended acategorial brain states may be noticed, monitored and/or tolerated by meditator-specific brain modules, involving mPFC and PCC, and the Frontal Negativity may be a correlate of the underlying processes.

### Summary

Due to evolutionary reasons the perceptual system is optimized to automatically shorten the unavoidable acategorial/unstable brain states at the transition from one stable perceptual brain state to the next to a minimum. During perceptual reversals of the ambiguous Necker lattice, however, such a transient acategorial brain state becomes temporally extended (plus about 50 ms, see Fig 2A and 2B), compared to “normal” transitions between perceptual states, as described in Kornmeier & Bach 2012 [47].

One effect of extensive meditation praxis may be the gain of volitional control over the phylogenetically evolved visual/perceptual automatisms. This may be reflected by activity in frontal control instances in the brain, monitoring and/or affecting even early, automatized sensory processing steps, as indicated by the present Frontal Negativity 160 ms after stimulus onset, probably originating from the mPFC and PCC. In the present study these meditation-specific control instances may be activated non-volitionally, by the extended acategorial perceptual brain state during a perceptual reversal of the Necker cube. Meditators may also be able to volitionally activate these control instances during meditation [128] and particularly if they experience altered states of consciousness. And we may be able to measure this in future studies.
